# Feasibility and safety of using local anaesthesia with conscious sedation during complex cardiac implantable electronic device procedures

**DOI:** 10.1038/s41598-018-25457-x

**Published:** 2018-05-08

**Authors:** Elif Kaya, Hendrik Südkamp, Julia Lortz, Tienush Rassaf, Rolf Alexander Jánosi

**Affiliations:** Department of Cardiology and Vascular Medicine, West German Heart and Vascular Center Essen, University of Duisburg-Essen, Essen, Germany

## Abstract

We assessed the feasibility and safety of using local anaesthesia with conscious sedation as an alternative to general anaesthesia during complex and noncomplex cardiac implantable device procedures. We enrolled 279 consecutive patients who underwent cardiac device implantation/replacement at our tertiary/quaternary cardiac specialist hospital during a 17-month study period. Continuous combined intravenous conscious sedation with propofol and midazolam plus fentanyl and local anaesthesia were used for all procedures. Among the patients, 113, 59, 43, and 64 patients underwent pacemaker implantation, implantable cardiac defibrillator implantation, cardiac resynchronisation therapy device implantation, and generator exchange, respectively. The procedural success rate was 100%, with no apnoea or hypoxia episodes requiring therapeutic intervention. None of the patients required conversion to general anaesthesia. The mean surgical duration was longer for complex vs. noncomplex procedures (p = 0.003). The minimum mean arterial pressure during complex procedures was slightly lower than that during noncomplex procedures (p = 0.03). The perioperative (<24 h) mortality rate was 0%, and neither complexity group required tracheal intubation. Only two patients (0.7%) required unplanned intensive care unit admission for further surveillance. Our findings suggest that local anaesthesia with conscious sedation is a safe and feasible option for cardiac device implantation procedures, including complex procedures.

## Introduction

Even complex cardiac implantable electronic device (CIED) procedures are increasingly performed using local anaesthesia with conscious sedation rather than general anaesthesia. Twenty years of experience supports the use of conscious sedation during the subpectoral implantation of simple cardiac devices. However, due to limited surgical centre experience, there is some concern regarding the use of this approach during the implantation of more-complex devices such as implantable cardiac defibrillators (ICDs) and cardiac resynchronisation therapy (CRT) devices. Respect for the mental and psychological status and comorbidities of the patient is the main reason for still using general anaesthesia in complex CIED procedures. But this approach is associated with increased risk owing to haemodynamic effects, particularly in elderly patients and patients with heart failure^1–11^. Conscious sedation is an attractive alternative, especially in tertiary/quaternary centres that provide care to such high-risk patients. However, there is some debate regarding the safety of using propofol because of possible undesirable side effects, including cardiovascular depression^4,6,12^.

The aim of this study was to summarise our experience employing local anaesthesia with conscious sedation for all noncomplex and complex CIED procedures at a single centre. Specifically, we assessed the feasibility, safety, and efficacy of local anaesthesia combined with conscious sedation using midazolam and propofol plus fentanyl.

## Methods

### Patients

We enrolled a consecutive series of patients who underwent CIED procedures at our hospital between January 2016 and April 2017 (Table 1). We recorded routinely collected data to evaluate the safety and efficacy of the procedures for all patients. The study was performed in accordance with the 1975 Declaration of Helsinki and approved by the s committee of the University of Duisburg-Essen (approval number: 17-7701-BO). After written informed consent was obtained from all patients, all parameters were entered into an internet-based electronic case report form by the centre. Patient records were de-identified and analysed anonymously.

**Table 1:** Baseline demographics: clinical data and characteristics.

Patient characteristic (N = 279)	
age (years)	70.8 ± 13.0
male, n (%)	185 (66.3%)
BMI	27.39 ± 5.03
congestive heart failure	
ischemic, n (%)	41 (14.6%)
non-ischemic, n (%)	38 (13.6%)
history of AF, n (%)	130 (46.5%)
left ventricular ejection fraction, (%)	38 ± 14
ASA II	40 (14.3%)
ASA III	170 (60.9%)
ASA IV	54 (18.3%)
hospital stay (days)	10.2 ± 8.2
postoperative stay (days)	5.7 ± 6.5
procedure time (min)	53.2 ± 42.1
CRP (mg/dl)	1.5 ± 2.6
WBC (/nl)	7.7 ± 3.8
Hb (g/dl)	12.9 ± 2.1

AF, atrial fibrillation; ASA, American Society of Anesthesiologists; BMI, body mass index; CRP, c-reactive protein; Hb, haemoglobin; WBC, white blood cells.

### Implantation procedures

All patients underwent standardised cardiac device implantation, replacement, or revision procedures under conscious sedation combined with local anaesthesia. The procedures included pacemaker implantation, ICD implantation for both primary and secondary prevention, subcutaneous ICD (s-ICD) implantation, CRT device implantation, and generator exchange procedures. We defined single- and dual-chamber pacemaker implantation and nonsubpectoral generator exchange as noncomplex procedures and all other CIED procedures as complex procedures, which are still frequently performed under general anaesthesia. Conscious sedation was achieved with fentanyl and a combination of midazolam and propofol.

The implantation procedures were performed in an electrophysiology laboratory with a complete anaesthetic infrastructure, including a ventilator and anaesthetic agents that were available in case of an emergency. The blood pressure of each patient was monitored throughout the procedure; measurements were checked against a baseline reference blood pressure at 3-, 5-, or 10-min intervals using a non-invasive Dinamap system. In patients with reduced cardiovascular function, blood pressure was continuously monitored using a radial artery catheter. Oxygen saturation was continuously monitored via pulse oximetry, and all patients received supplemental oxygen either by a mask or nasal cannula. Oxygen was continuously supplemented at 2–6 L/min to maintain an oxygen saturation of >95%. An experienced anaesthesiologist or cardiologist with experience in intensive care medicine was present during all procedures. In addition, two nurses with training and experience in airway management and advanced life support were present. During each procedure, at least one member of the staff was exclusively responsible for close monitoring of the patient. All procedures were performed by a single experienced operator (E.K.).

Prophylactic antibiotic therapy (cephazolin) was provided 30 min prior to surgery. Local anaesthesia was induced with 20–40 mL of Scandicain 1%; this facilitated venous access, pocket formation, and lead placement. The dose was determined at the discretion of the operator. Pre-drawn flumazenil and naloxone were always available for the immediate reversal of sedation if necessary. The standard procedure involved access to the cephalican vein. If hypotension occurred during the procedure, initial treatment with intravenous saline infusion was initiated. If the systemic pressure failed to increase, intravenous catecholamines were administered. The duration of the procedure was defined as the time from the first incision placement to the last skin suture. Tight banding was performed in all patients for 24 h after the procedure. All patients were observed in the catheter laboratory recovery area until they were fully awake. Subsequently, they were transferred back to the general ward.

During the study period, there were no changes in the technique of implantation. All patients met the appropriate criteria for permanent pacemaker, ICD, or CRT device implantation^13^.

### Conscious sedation

We defined conscious sedation as a moderate level of sedation that provided the drug-induced depression of consciousness while preserving respiratory function and maintaining patient responsiveness. Conscious sedation was achieved with a combination of midazolam and propofol with fentanyl administered intravenously at the start of the procedure. The standard initial dosages were 2–5 mg for midazolam, 30 mg for propofol, and 30–50 μg of fentanyl. Patients > 75 years of age and weighing < 70 kg received an initial midazolam dose of 1–2 mg. Additional doses to maintain adequate sedation were titrated during the procedure when necessary. The cumulative dose of all agents was calculated and documented for each patient.

### Statistical analysis

All procedural and anaesthetic data, including demographic and outcome data, were entered into a database. When a patient required a cardioactive agent, the occurrence of hypotension or desaturation and the conversion of sedation to general anaesthesia were documented. Other parameters, including the procedure time, sedative and analgesic dosages, and requirement for respiratory support, were also recorded. Patient tolerability toward the procedure was additionally assessed. Hypotension was defined as a decrease in the systolic blood pressure of ≥ 30% from baseline or a mean arterial pressure (MAP) of < 65 mmHg for more than 3 min. All statistical analyses were performed using SPSS version 24.0 (IBM SPSS, Chicago, IL, USA). Continuous variables are expressed as means and standard deviations and categorical variables are expressed as numbers and percentages. With regard to normally distributed variables, comparisons between groups were performed using Student’s *t*-tests for continuous variables and *χ*^2^ tests for categorical variables. With regard to non-normally distributed continuous variables, comparisons between groups were performed using Mann–Whitney U tests. For all analyses, differences were considered statistically significant at p < 0.05.

### Data availability statement

Data will be provided on request.

## Results

We enrolled 279 consecutive patients (185 men; mean age, 70.8 ± 13.0 years) in this study. Among these, 113, 59, 43, and 64 patients underwent pacemaker implantation, ICD implantation (including three s-ICD implantations), CRT device implantation, and generator exchange, respectively. Regarding device implantation, 199 of the devices were implanted subfascially (71.3%), 73 were implanted subpectorally (26.2%), and two were implanted subcutaneously (0.7%). Among the patients who underwent generator exchange (23.0%), the majority of the procedures were subpectoral (40 patients, 63.5%).

Women required significantly higher doses of fentanyl for analgesia than did men (61.0 ± 70.4 μg vs. 42.7 ± 50.2 μg, p = 0.012), but no differences in the procedural duration were noted between women and men (52.7 ± 33.4 min vs. 53.5 ± 46.0 min, p = 0.4).

CRT device implantations took longer than did single- and dual-chamber device implantations (107.3 ± 66.8 min vs. 49.2 ± 33.9 min, p < 0.0001), but no difference in the mean dose of fentanyl was observed between these procedural groups (67.5 ± 59.2 μg vs. 38.9 ± 41.4 μg, p = 0.972). However, CRT device implantation required higher cumulative doses of midazolam and propofol than did single- and dual-chamber device implantation (midazolam: 4.8 ± 3.7 mg vs. 2.3 ± 2.3 mg, p = 0.001; propofol: 34.2 ± 63.2 mg vs. 9.5 ± 30.3 mg, p = 0.027). Patients who underwent pacemaker or ICD implantation received lower doses of propofol than did those who underwent CRT device implantation, predominantly because of a longer mean procedural duration. Procedural success was achieved in all patients.

**Figure 1:**
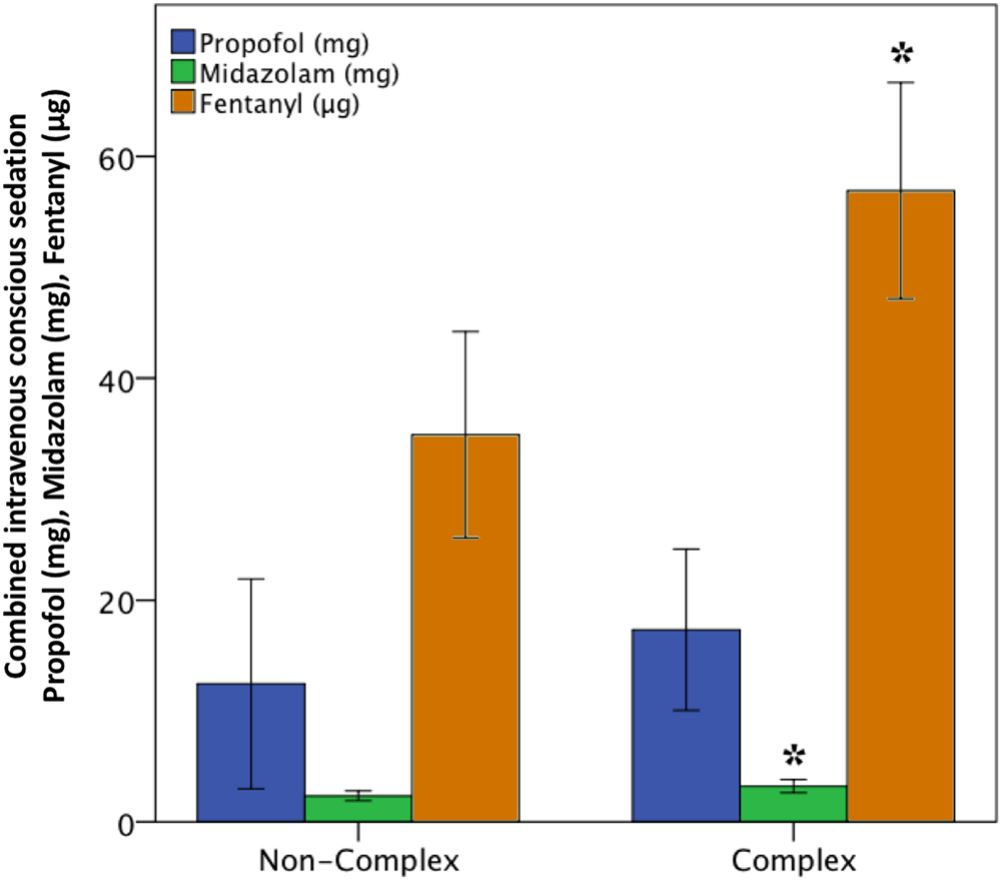
Mean dosages of propofol, midazolam, and fentanyl used during noncomplex and complex procedures. The error bars represent the standard error of the mean (2 SE). *p < 0.05.

**Figure 2:**
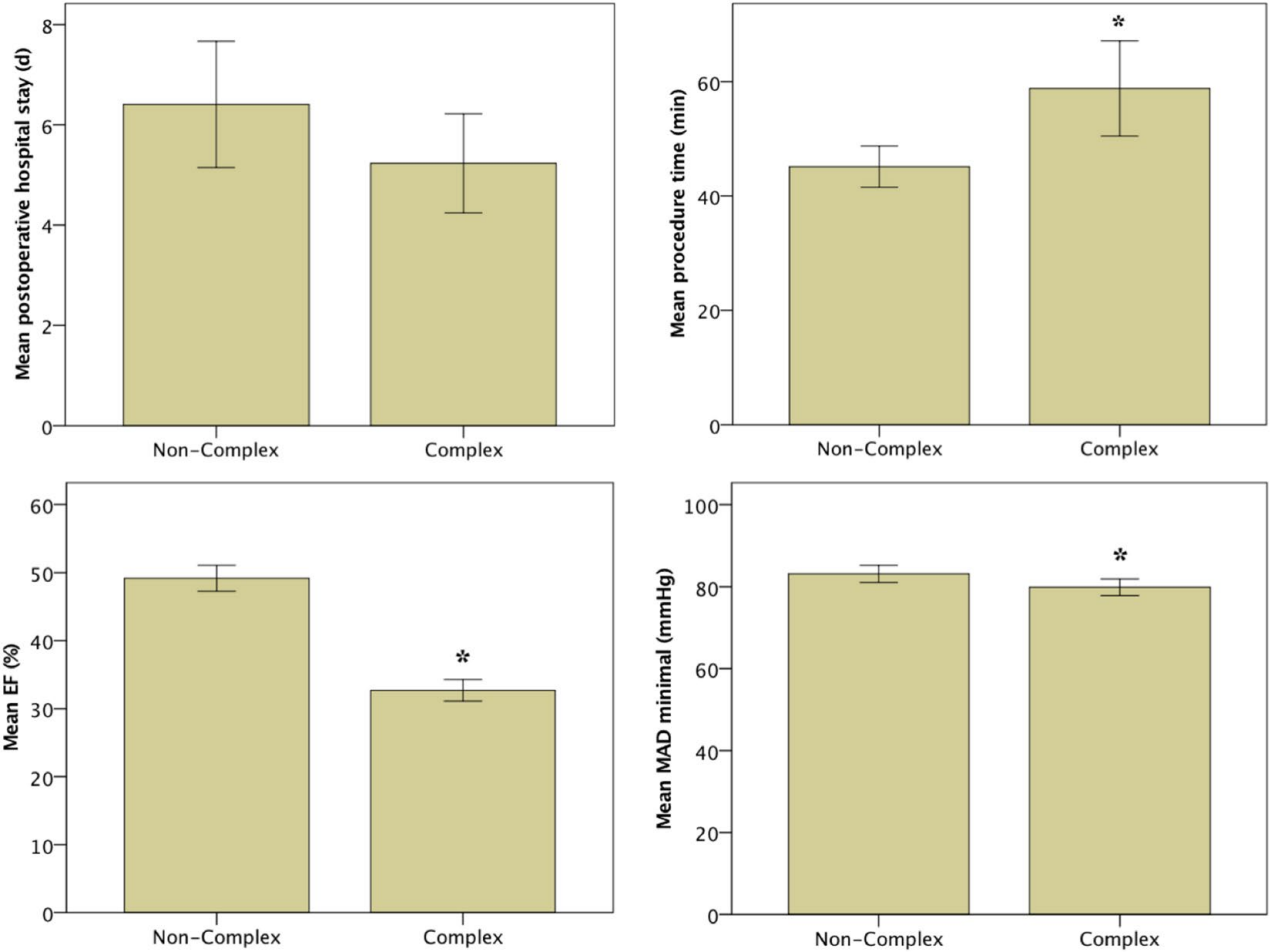
Procedural details for noncomplex and complex cardiac implantable electronic device procedures. Despite a significantly longer procedure time and significantly lower ejection fraction (EF) in the complex group, the minimal mean arterial pressure (MAP) and postoperative stay are comparable between the two groups. The error bars represent the standard error of the mean (2 SE). *p < 0.05.

We also compared noncomplex and complex procedures, the latter of which are still frequently performed under general anaesthesia. In both groups, no episodes of apnoea or hypoxia requiring therapeutic intervention during conscious sedation and local anaesthesia were observed. The mean procedural duration was longer in the complex group than it was in the noncomplex group (p = 0.003; Table 2). The average sedation dosages for fentanyl and midazolam were higher during complex procedures than they were during noncomplex procedures (fentanyl: p = 0.002; midazolam: p = 0.03; Fig. 1, Table 2), whereas no difference in the mean dosage of propofol was found between the two complexity groups (p = 0.403; Table 2). Moreover, no differences in the length of postoperative hospital stay (p = 0.136) and MAP decrease (p = 0.853) were noted between the complexity groups (Fig. 2, Table 2). The minimum MAP during complex procedures was only slightly lower than was that during noncomplex procedures (p = 0.03; Fig. 2, Table 2). The ejection fraction in the complex group was lower than was that in the noncomplex group (p < 0.0001; Fig. 2, Table 2).

**Table 2:** Sedation use and dosage by procedure type.

	Non-complex (N = 119)	Complex (N = 160)	p-value
age	74.9 ± 12.0	68.3 ± 12.3	**0.0001**
BMI	26.7 ± 4.6	27.9 ± 5.3	0.067
postoperative stay (days)	6.4 ± 6.8	5.2 ± 6.2	0.136
EF (%)	49 ± 10	32 ± 10	**0.0001**
procedure time (min)	45.1 ± 19.3	58.8 ± 52.2	**0.003**
MAP initial (mmHg)	105.3 ± 12.1	100.9 ± 13.5	**0.01**
MAP minimal (mmHg)	83.1 ± 10.7	79.9 ± 12.3	**0.03**
MAP decrease (%)	20.6 ± 9.0	20.4 ± 9.8	0.853
creatinine (mg/dl)	1.38 ± 1.0	1.38 ± 0.8	0.981
postoperative creatinine (mg/dl)	1.39 ± 1.2	1.47 ± 1.1	0.640
Hb (mg/dl)	13.3 ± 2.0	13.7 ± 1.9	0.078
Hb difference (mg/dl)	0.02 ± 1.3	0.40 ± 1.0	**0.02**
average sedation dosage			
Fentanyl dosage (μg)	34.9 ± 48.2	56.4 ± 59.4	**0.002**
Propofol (mg)	12.3 ± 49.0	17.2 ± 44.3	0.403
Midazolam (mg)	2.4 ± 2.3	3.2 ± 3.6	**0.03**

ASA, American Society of Anesthesiologists; BMI, body mass index; EF, ejection fraction; Hb, haemoglobin; MAP, mean arterial pressure.

In 44 patients (16%), the MAP decreased by 34.6 ± 9.7 mmHg (vs. 17.9 ± 6.7 in the normotensive group, p = 0.0001) from an initial value of 105.1 ± 18.0 mmHg. This decrease was primarily managed conservatively by the administration of intravenous saline. Pharmacological interventions were only necessary for 12 patients (4.3%), who were safely managed with intravenous saline and incremental boli of phenylephrine (100 cg), epinephrine (10 μg), or norepinephrine (10 μg). Inotrope use did not differ between the noncomplex and complex groups (3.3% vs. 5.0%, respectively, p = 0.568).

All patients were discharged a mean of 5.7 ± 6.5 days after implantation. All devices were functional during predischarge testing. No cases of perioperative (<24 h) mortality were observed. Two patients (0.71%) required unplanned intensive care unit admission for further surveillance.

## Discussion

The majority of endoscopic, dental, and ureteroscopic procedures are already being performed under conscious sedation in the absence of an anaesthesiologist^14–17^, and several studies have reported using midazolam for conscious sedation during a number of procedures performed in cardiac electrophysiology laboratories^7^. Sedation with midazolam is usually operator-guided and has been demonstrated as safe and effective for several different procedures^3^. In recent years, this approach has been similarly implemented for pacemaker implantation^8^. However, there is some hesitation regarding the use of local anaesthesia with conscious sedation for complex CRT device implantation procedures or s-ICD implantation procedures requiring subcutaneous leads. For one, CRT device implantations take longer than do pacemaker or ICD implantations. The procedural duration is another important consideration for these procedures. Furthermore, implantations into a submuscular pocket are considered potentially painful procedures that warrant the use of general anaesthesia^18^. Hence, it is unclear whether local anaesthesia with conscious sedation would be effective in such cases.

On the other hand, general anaesthesia increases the risk of laryngeal damage, cardiovascular complications, and pneumonia, particularly in elderly populations with impaired left ventricular function and comorbidities that are typical of patients admitted to tertiary/quaternary hospitals^1–11,19^. Moreover, the utilisation of anaesthesiology staff and services can be costly and requires exhaustive co-ordination, and the use of general anaesthesia can indirectly increase patient costs by prolonging the procedural duration and length of hospital stay.

### Pain

Postoperative and procedural self-reported pain may be more severe among women than among men^20^, and the female sex has been more frequently associated with moderate-to-severe pain during the early postoperative period compared with the male sex^10^. Although we did not evaluate the postoperative period in our analysis, we found that higher analgesic doses were required for women than for men, and this was consistent with previous findings^10^.

### Staff

In the present study, all noncomplex and complex device implantations were safely and successfully performed under local anaesthesia by experienced staff. For the safe performance of these procedures, a well-trained staff member with experience in airway management and an appropriate catheter/electrophysiology laboratory infrastructure are required. A benefit of using local anaesthesia under conscious sedation is the nonrequirement of a complete anaesthetic team. Nevertheless, we ensured that an experienced anaesthesiologist or cardiologist with experience in intensive care medicine was present during all procedures, in addition to two nurses with training and experience in airway management and advanced life support. Thus, the presence of at least one individual who was exclusively responsible for close monitoring of the patient was ensured.

### Safety

The necessity of general anaesthesia for cardiac device implantation remains a controversial topic^4,5^. In our consecutive cohort, no deaths or circumstances necessitating intubation or formal ventilatory support associated with the use of local anaesthesia and conscious sedation were observed. The safety and convenience of device implantation that we noted using our approach are consistent with those found in some previous reports^2–4,6–8,11^. A large single-centre study found that intravenous sedation could be safely administered in the absence of an anaesthesiologist^9^. We demonstrated that even complex procedures, such as CRT device and s-ICD implantations, are feasible under conscious sedation. One previous study reported hypotension and the need for pharmacological support in approximately 50% of patients who received general anaesthesia^2^. Another study similarly reported a hypotension incidence of up to 56% in patients undergoing CRT device implantation under conscious sedation, with a higher incidence in patients with a low ejection fraction^12^. This was likely related to the dosage of propofol. At our institution, we use a combination of midazolam and propofol for sedation to avoid cardiovascular complications^17^. Using this combination, a similar dosage of propofol was required for both complex and noncomplex procedures (17.2 ± 44.3 vs. 12.3 ± 49.0, respectively, p = 0.403). In addition, hypotension occurred more frequently in patients receiving general anaesthesia than it did in those receiving local anaesthesia with conscious sedation for the implantation of a biventricular pacing device^8^. Nevertheless, the authors described an increase in the use of general anaesthesia over the 7-year study period owing to the lack of changes in the medical personnel involved and fear of the need for eventual conversion to general anaesthesia in some patients^8^. Here, none of the patients required conversion to general anaesthesia. Collectively, our results support previous findings documenting the safety and feasibility of using local anaesthesia with conscious sedation for cardiac device implantation.

### Limitations

The present study has several limitations. First, it was limited by the retrospective design and therefore a comparative analysis of local anaesthesia with or without sedation was not available. Further studies should also include randomized trials about local anesthesia with or without sedation. Second, it was performed in a single tertiary/quaternary cardiology department in a university hospital in Germany; therefore, the demographic characteristics of our cohort may not be representative of the general population and were likely biased by the large proportion of high-risk patients.

## Conclusions

To our knowledge, this is the first single-centre study to document the use of local anaesthesia with conscious sedation during various noncomplex and complex cardiac device implantation procedures. The results suggest that our anaesthetic protocol is both safe and feasible. In particular, the use of local anaesthesia with conscious sedation as an alternative to general anaesthesia offers advantages for tertiary/quaternary hospitals that treat high-risk patients who generally exhibit poor left ventricular function and/or comorbidities such as ischaemic heart disease and ventricular arrhythmia. We believe that, in place of an anaesthesiologist, a highly experienced operator together with a cardiologist trained in intensive care are indispensable, particularly for procedures in elderly patients. However, future prospective trials are necessary to confirm our findings.
